# Diversity, Ecology and Biogeochemistry of Cyst-Forming Acantharia (Radiolaria) in the Oceans

**DOI:** 10.1371/journal.pone.0053598

**Published:** 2013-01-11

**Authors:** Johan Decelle, Patrick Martin, Katsiaryna Paborstava, David W. Pond, Geraint Tarling, Frédéric Mahé, Colomban de Vargas, Richard Lampitt, Fabrice Not

**Affiliations:** 1 CNRS, UMR 7144, EPPO, Université Pierre et Marie Curie, Station Biologique de Roscoff - Place Georges Teissier, Roscoff, France; 2 National Oceanography Centre, Southampton, United Kingdom; 3 British Antarctic Survey, Cambridge, United Kingdom; Royal Netherlands Institute of Sea Research (NIOZ), The Netherlands

## Abstract

Marine planktonic organisms that undertake active vertical migrations over their life cycle are important contributors to downward particle flux in the oceans. Acantharia, globally distributed heterotrophic protists that are unique in building skeletons of celestite (strontium sulfate), can produce reproductive cysts covered by a heavy mineral shell that sink rapidly from surface to deep waters. We combined phylogenetic and biogeochemical analyses to explore the ecological and biogeochemical significance of this reproductive strategy. Phylogenetic analysis of the 18S and 28S rRNA genes of different cyst morphotypes collected in different oceans indicated that cyst-forming Acantharia belong to three early diverging and essentially non symbiotic clades from the orders Chaunacanthida and Holacanthida. Environmental high-throughput V9 tag sequences and clone libraries of the 18S rRNA showed that the three clades are widely distributed in the Indian, Atlantic and Pacific Oceans at different latitudes, but appear prominent in regions of higher primary productivity. Moreover, sequences of cyst-forming Acantharia were distributed evenly in both the photic and mesopelagic zone, a vertical distribution that we attribute to their life cycle where flagellated swarmers are released in deep waters from sinking cysts. Bathypelagic sediment traps in the subantarctic and oligotrophic subtropical Atlantic Ocean showed that downward flux of Acantharia was only large at high-latitudes and during a phytoplankton bloom. Their contribution to the total monthly particulate organic matter flux can represent up to 3%. High organic carbon export in cold waters would be a putative nutritional source for juveniles ascending in the water column. This study improves our understanding of the life cycle and biogeochemical contribution of Acantharia, and brings new insights into a remarkable reproductive strategy in marine protists.

## Introduction

The dark ocean, the largest habitat in the biosphere [1], is of major importance in global ocean biogeochemistry. Spanning the mesopelagic (200–1000 m) and bathypelagic (1000–4000 m) zones, the dark ocean is a key reservoir of organic carbon that is produced in the photic layer and sinks downward in form of particles. Large particles, such as fecal pellets and phytodetrital aggregates, but also some unicellular planktonic organisms, such as Radiolaria, are major contributors to the downward particle organic carbon (POC) flux [2]. Among the Radiolaria, Acantharia have been shown to contribute to mesopelagic and bathypelagic POC flux via cyst formation [3], [4], thus playing an important role in the biological pump of carbon.

Acantharia are cosmopolitan and abundant protists, and are the only organisms known to form entire mineral skeletons of celestite (strontium sulfate, SrSO_4_). They also have the capacity to completely change their morphology over the course of their life cycle in order to form cysts. Unlike the cysts of other planktonic single-celled organisms, such as dinoflagellates, diatoms or ciliates, which are resting stages produced to overcome unfavorable environmental conditions [5], [6], [7], acantharian cysts are directly linked to reproduction [8], [9], [10], [11]. Thousands of flagellated cells (∼2–3 µm [12]), called swarmers, are formed within each cyst and released either through pores or upon rupture of the cyst wall. However, some acantharian species do not form cysts and in this case, swarmers are produced directly through the vegetative stage (adult form) without encystment.

During cyst formation, the spicules of the skeleton and the main cellular components (myonemes, axopods, ectoplasm etc.) are resorbed. The cell loses its buoyancy and starts depositing a robust seed-like celestite shell of 5–7 µm in thickness, generally bigger than the vegetative cell, up to 1 mm [9], [11]. Cysts exhibit a large variety of shape and forms, presumably produced by distinct acantharian families and species: oval, round, elongated, with or without mineral plates and pores etc. Some of them have been described in the past as new acantharian families and species [13] or classified according to their form (e.g. forma Allas, Folium, Bimamma, Ampulla, Olive etc [14]). However, since the morphological characters used for species identification completely disappear during encystment, and cyst formation is very rarely observed upon collecting adult stages, it is not clear which taxonomic groups are able to encyst. DNA identification could therefore be a useful tool to unambiguously assign certain types of cyst to adult morphospecies within molecular-based clades, and allow taxonomic distinction between cyst-forming and non-cyst-forming Acantharia. A recent molecular phylogeny of Acantharia described at least nine molecular clades (I, III, IV and A to F). Clades A, B and D represent the taxonomic order Holacanthida, and the most recently diverging clades, E and F, are composed of the orders Symphiacanthida and Arthracanthida. The order Chaunacanthida is only found in clade C. Clades I, III and IV are only composed of environmental sequences, from which the morphology and taxonomy are unknown [15].

Although Acantharia are mostly found in the surface ocean, numerous environmental DNA sequences (18S rRNA gene) have been found deeper in the mesopelagic and bathypelagic zones [16], [17], [18], [19], [20]. The deep sequences probably originate at least partly from sinking cysts and release of swarmers. Ballasted with the mineral celestite, which is about twice as dense as the calcium carbonate of which other protistan tests are built (SrSO_4_ density = 3.96 g cm^−3^), acantharian cysts rapidly sink from the surface to depth. The largest specimens can sink up to 500 m day^−1^ [4], and presumably release swarmers far below the surface. Acantharian cysts have been collected in sediment traps and net samples around the world, at high latitudes (Antarctic, East Greenland Sea, Iceland Basin, [3], [4], [21]), and in tropical and subtropical waters (the Mediterranean Sea, the Atlantic, Indian and Pacific Oceans, [8], [11], [22]).

Acantharia contribute to the downward POC flux, which is one important factor regulating atmospheric CO_2_ concentration [23]. Celestite is highly soluble in seawater and the cyst shell appears to dissolve quickly once cysts start sinking [3], [24]. Most previous studies of acantharian fluxes, focusing mainly on low latitudes, have hence concluded that cysts contribute significantly to shallow POC fluxes, but not appreciably below the upper 200–300 m [3], [21], [25]. However, a particularly large type of acantharian cyst was recently found in sediment traps from 2000 m in the Iceland Basin, contributing up to 48% of POC flux during a 2-week sampling interval [4]. It was hypothesized that deep sedimentation of cysts might be limited to high latitudes and exhibit a seasonal pattern, where high primary productivity during seasonal phytoplankton blooms triggers encystment such that juvenile Acantharia at depth can exploit the subsequent pulse of sinking phytodetritus as a food source upon bloom collapse [4]. However, a more thorough study of the contribution of Acantharia to deep particle fluxes, and its latitudinal variability, has not yet been undertaken. Moreover, the biogeography and vertical distribution of the vegetative stage of cyst-forming Acantharia needs closer examination to understand how their ecology and life cycle influence the downward POC and strontium fluxes.

Beyond implications for carbon biogeochemistry, Acantharia affect the oceanic cycling of strontium and barium, which are highly concentrated in cyst shells [3], [26]. By precipitating celestite, Acantharia create vertical and horizontal gradients in strontium concentration [3], [27], [28]. Depletion of strontium in seawater due to Acantharia is sometimes claimed to be problematic for the use of strontium/calcium ratios in corals and planktonic foraminifera to reconstruct past sea-surface temperatures [28]. While its role remains enigmatic, strontium is also required for calcification in many marine organisms [29], [30]. This calls for more examination of acantharian ecology and biogeochemical flux inherent to the life cycle.

Here, we used molecular tools to genetically identify cyst-forming Acantharia, and investigate their vertical distribution in different oceanic regions worldwide through the V9 region of the 18S rRNA gene. Finally, we assessed the biogeochemical contribution and seasonality of cyst sedimentation by measuring strontium fluxes at four sites in the Atlantic Ocean.

## Materials and Methods

### Collection of cysts

**Figure 1:**
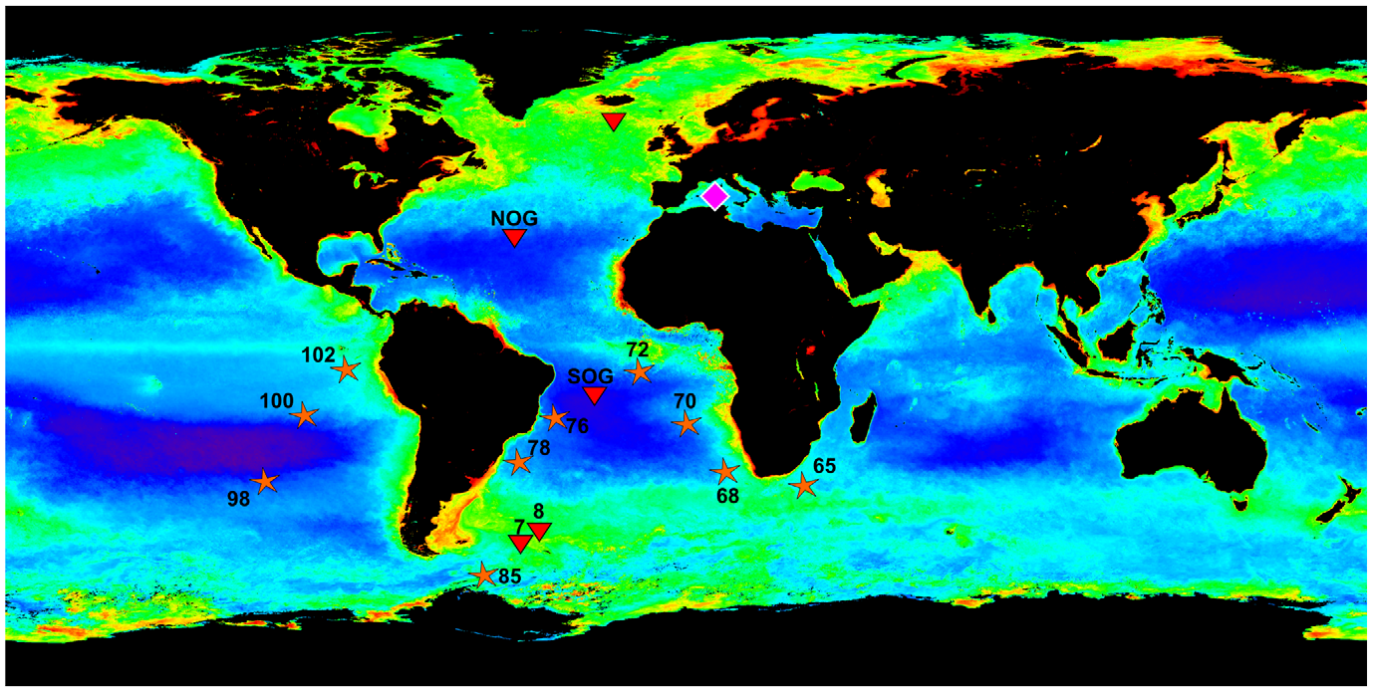
Sampling sites. Map of annual integrated chlorophyll concentration (high values are indicated in green and red) showing the sampling locations of acantharian cysts from meso-and bathypelagic traps (red triangle), and surface plankton nets (pink diamond). Genetic sequences (V9 region of the 18Sr RNA gene) have been also obtained from the surface and mesopelagic (orange star). (Map obtained from OceanColor website: http://oceancolor.gsfc.nasa.gov/).

Cysts were collected in distinct oceanic regions. Some cysts were isolated from plankton net tows between 0 and 50 m in the Mediterranean Sea (Villefranche-sur-mer; Table 1) in 2010 and 2011. They were individually photographed and placed in a guanidine-containing extraction buffer (GITC). Additional cysts were collected during 2006–2008 in the Iceland basin, either at 160 and 620 m in 3-day deployments of neutrally buoyant PELAGRA sediment traps [31], or at 2000 m using year-long deployments of bottom-tethered sediment traps [4]. A few adult cells (vegetative stage) were also found in the PELAGRA traps. All trap samples were fixed in formaldehyde preservative (see below). Cysts and adults were individually picked from the three depths, washed several times in PBS buffer to remove the formaldehyde, isolated in GITC buffer and preserved at −20°C before processing. All sampling locations are shown in Figure 1.

**Table 1:** Sampling sites and equipment used.

Oceanic region	Sampling sites	Latitude	Longitude	Date	Sampling	Depth (m)
Mediterranean Sea	Villefranche-sur-mer	43°40.552 N	7°18.447 E	Nov-10	Net samples	0–50 m
North Atlantic Ocean	Iceland basin	60°N	20°W	Nov 06–July 07	Trap samples	2000
North Atlantic Ocean	Iceland basin	61°N	26°W	May-08	Trap samples	160–620
North Atlantic Ocean	NOG	23°46.119 N	41°5.419 W	Nov 07–Nov 09	Trap samples	3000
South Atlantic Ocean	SOG	18°31.48 S	25°6.0 W	May 08–June 10	Trap samples	3000
South Atlantic Ocean	St 7	55°15.320 S	41°21′.273 W	Mar 08–Dec 08	Trap samples	2000
South Atlantic Ocean	St 8	52°48.288 S	40°6.522 W	Feb 08–Dec 08	Trap samples	2000
Indian Ocean	Station 65	35°45.299 S	26°19.199 E	Jul-10	Niskin bottles	0 and 800
South Atlantic Ocean	Station 68	31°58.416 S	5°21.612 E	Sep-10	Niskin bottles	0 and 700
South Atlantic Ocean	Station 70	18°4.229 S	4°54.82 W	Sep-10	Niskin bottles	0 and 800
South Atlantic Ocean	Station 72	8°13.415 S	17°5.202 W	Oct-10	Niskin bottles	0 and 850
South Atlantic Ocean	Station 76	20°0.78 S	35°43.296 W	Oct-10	Niskin bottles	0 and 800
South Atlantic Ocean	Station 78	30°47.629 S	43°41.878 W	Nov-10	Niskin bottles	0 and 800
Antarctic	Station 85	62°51.41 S	49°41.817 W	Jan-11	Niskin bottles	0 and 800
South Pacific Ocean	Station 98	25°6.431 S	111°20.25 W	Apr-11	Niskin bottles	0 and 485
South Pacific Ocean	Station 100	13°59.394 S	96°51.343 W	Apr-11	Niskin bottles	0 and 177
South Pacific Ocean	Station 102	5°44.135 S	85°46.669 W	Apr-11	Niskin bottles	0 and 480

No specific permits were required for the field sites, as the location is not privately-owned or protected in any way (international oceanic waters), and the studied organisms did not involve endangered or protected species.

### DNA extraction, amplification and phylogenetic analyses

DNA extraction was carried out on each isolated cyst as previously described [15]. Single-cell Polymerase Chain Reaction (PCR) was used to amplify 18S and 28S (D1 and D2 regions) ribosomal DNA markers with Radiolaria-specific primers (see 15 for primer details). Following amplification, the PCR products were purified by EXOSAP-IT (GE Healthcare Bio-Sciences Corp.), and bidirectionally sequenced using the ABI-PRISM Big Dye Terminator Cycle Sequencing Kit (Applied Biosystems). Accession numbers of the 18S and 28S rRNA sequences of cysts are given in Table S1.

The matrices of 18S rRNA and partial 28S rRNA sequences were individually aligned with Muscle, implemented in Seaview [32]. As the topology of 18S and 28S rRNA genes are congruent [15], the two aligned matrices were concatenated with Sequence Matrix [33], forming a 2386 bp-long dataset containing 104 taxa. The GTR+G+I model of nucleotide substitutions was selected by MEGA 5 [34] according to the BIC and AIC criteria. The phylogenetic inference by Maximum Likelihood (ML) was then performed with PhyML v3.0 [35], and resampled 500 times by non-parametric bootstrapping. Bayesian inference was conducted on the concatenated dataset using Beast v.1.6.1 and companion software [36] under the GTR model taking into account 4-class gamma and invariant sites. Two Markov Chain Monte Carlo (MCMC) chains were run for 30 million generations, sampling every 1000 generations. The two runs were combined with LogCombiner v1.6.1, and convergence of log-likelihoods and parameter values were assessed in Tracer v1.4.1. 10% of the total trees were discarded as burn-in, and the remaining trees were used by TreeAnnotator 1.5.4 to build the consensus tree and to calculate the posterior probabilities (PP) of each node.

### Biogeography and diversity of Acantharia in the photic and mesopelagic zones

#### Clone libraries from previous environmental surveys

All the 18S rRNA environmental sequences from clone libraries (Sanger technology) related to Acantharia (*n* = 260) were retrieved from GenBank version 190 in March 2012. Any associated information for each sequence, such as location and depth of sampling was recorded. We used pplacer to compare the phylogenetic relationships between the environmental sequences and cyst sequences [37] with the 18S rRNA gene reference alignment. The advantage of using this program is that all the 18S rRNA environmental sequences can be comprehensively analyzed whatever their length and region. The 260 environmental sequences (query sequences) were aligned with a Hidden Markov Model built from the reference alignment using tools from the HMMER v3.0 suite (http://hmmer.org/), with default parameters. Using this alignment and the inferred reference tree, pplacer determined the most probable location for each query sequence and represented them as additional branches in the tree.

### V9 tag sequences of the 18S rRNA and taxonomic assignation

In the mesopelagic zone, seawater samples were collected with Niskin bottles at different locations in the South Indian, Atlantic and Pacific oceans during the TaraOceans expedition ([38], details in Table 1 and Figure 1). Around 90 liters of seawater were filtered onto consecutive 3-µm and 0.8-µm mesh polycarbonate membranes (diameter: 142 mm). At the same locations, samples were also collected in the photic zone (surface and Deep Chlorophyll Maximum, DCM) by pumping water and towing plankton nets (20-µm and 180-µm mesh size). Pumped water was filtered through a 5-µm and 0.8-µm membrane (0.8–5-µm size fraction), and net samples were filtered on a 20-µm membrane filter (20–2000-µm size fraction). All filters were subsequently preserved in liquid nitrogen.

DNA extraction was performed using the Nucleospin® DNA II kit. The V9 hypervariable region of the 18S rRNA gene was PCR amplified with general-eukaryote primers (1389F/1510R from [39]) with the Phusion® High-Fidelity DNA Polymerase (Finnzymes), purified using the NucleoSpin® Extract II kit (Macherey-Nagel, Hoerdt, France), and sequenced from both sides using the Illumina technology. The quality of the V9 tag sequences was screened following different steps. Only sequences that have exact forward and reverse primer match were considered. In addition, based on the list of quality values at each position, sequences with more than 1% of error in any 50 bp-section were discarded. Chimeras were then checked on the filtered sequences using the Usearch program [40].

The V9 reads were assigned based on a reference database of V9 sequences of Acantharia [15] and eukaryotes [41]. Each environmental sequence was compared to all reference sequences using an exact global pairwise alignment algorithm [42], and received the taxonomic assignation of its nearest-neighbor in the reference database (or of the last common ancestor in case of a tie). Pairwise alignments were computed with ggsearch, a tool from the version 36 of the FASTA program package [43], using default parameters, and results were stored in a sqlite database. Alignments containing both reference sequences and the assigned environmental V9 sequences were visually checked. Only reads assigned with more than 85% of identity with a reference sequence were considered in this study since below this value the V9 reads exhibit numerous errors randomly distributed along the sequence, including in well-conserved regions. V9 reads of Acantharia have been submitted to the Sequence Read Archive (SRA) in the EBI database under the study accession number ERP002023 (http://www.ebi.ac.uk/ena/data/view/ERP002023).

### Strontium measurements in sediment trap samples

In addition to the Iceland Basin sediment traps from which we isolated Acantharia (described above), we measured dissolved Sr in the preservative solution of bathypelagic time-series sediment traps deployed at four locations in the subtropical and subantarctic (Scotia Sea) Atlantic Ocean. Since celestite is highly soluble and dissolves upon collection in sediment traps, the preservative solution of trap samples containing Acantharia becomes highly elevated in Sr [4]. Since no other organisms are known to precipitate Sr as a major skeletal constituent, very high Sr concentrations in sediment trap preservatives would unambiguously identify a contribution by Acantharia to the particle flux.

Deployment locations, depths, and collection periods for all traps, including the Iceland Basin ones, are listed in Table 1. Sr fluxes collected by the Iceland Basin traps were measured previously and are already published [4], [31]. The subtropical and Scotia Sea traps were McLane Parflux Mark78H traps with 21 collection bottles, and were deployed at 2000 m (Scotia Sea) and 3000 m (subtropical). Collection bottles on the subtropical traps were filled with formaldehyde brine prior to deployment (2% formaldehyde with 0.5% w/v NaCl in seawater, buffered with 250 mg L^−1^ sodium tetraborate), those on the Scotia Sea traps with mercuric chloride-poisoned seawater. Upon recovery of the traps, samples were stored at 4°C.

Aliquots of preservative solution from the collection bottles were diluted to between 1∶500 and 1∶4000 by volume with 3% sub-boiled HNO_3_, spiked with 5 ng g^−1^ of indium (In) and rhenium (Re) as internal standards, and Sr and Ca concentrations measured on a Thermo X series inductively coupled plasma mass spectrometer. The instrument was calibrated with mixed Ca+Sr standards containing 5 ng g^−1^ In and Re.

Sr fluxes into collection bottles with elevated Sr concentration were calculated after subtracting the background Sr concentration (7.24–8.42 µg mL^−1^, depending on trap) by multiplying by the bottle volume (300 mL or 500 mL, depending on location) and dividing by the trap collection area (0.66 m^−2^) and by the collection period (14–31 days). The background concentration of Sr in seawater is around 8 µg mL^−1^, and in the sediment trap preservative samples was mostly between 7.1 and 8.4 µg mL^−1^. We hence calculated the background Sr concentration in the samples as the mean concentration of all bottles containing less than 9 µg mL^−1^ Sr, and considered samples to be significantly elevated in Sr if their concentration was higher than mean+2 times standard deviation of the bottles with <9 µg mL^−1^. While the cut-off of 9 µg mL^−1^ is somewhat arbitrary, this is in practice not relevant, since even a modest Sr flux would elevate the preservative concentration by several µg mL^−1^ above the background.

Uncertainties for the Sr measurements were propagated by assuming 1% analytical uncertainty in each Sr measurement [4] and from the calculated standard deviation of the Sr concentration in bottles with <9 µg Sr mL^−1^, i.e. the standard deviation of the background concentration.

## Results

### Molecular identification of acantharian cysts

**Figure 2:**
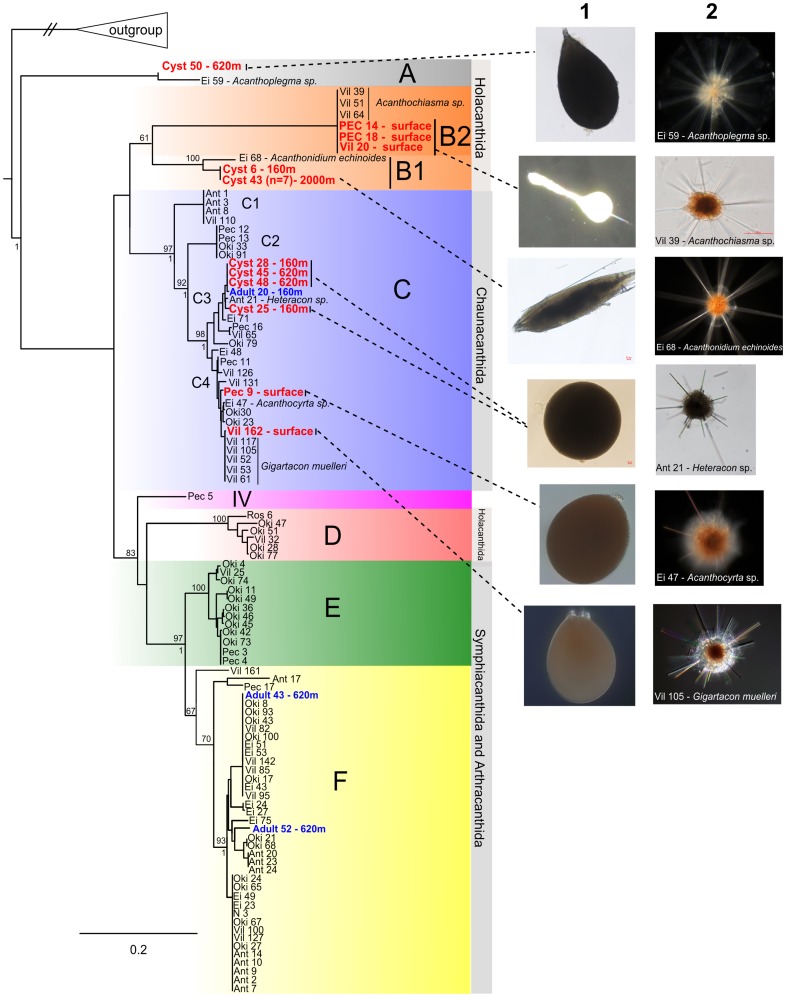
Phylogenetic placement of the cyst-forming Acantharia. Molecular phylogeny obtained with Maximum Likelihood analysis based on a concatenated matrix of 18S and 28S rRNA sequences of Acantharia (104 taxa and 2386 bp-long, GTR+I+G model), including cysts (bold red font) and vegetative (bold blue font) specimens collected in plankton nets and sediment traps at different depths. Each picture of a cyst (column 1) is shown together with the vegetative stage (column 2) that shares the highest genetic similarity. PhyML bootstrap percentages based on 500 pseudo-replicates and Bayesian posterior probabilities (PP) are indicated at each node (above and below, respectively) when support values were higher than 60% and 0.8, respectively. Species name of each taxon and accession numbers are provided in Table S1.

We obtained ten 18S and eleven 28S rRNA gene sequences from three distinct morphotypes of acantharian cysts (pear-shaped, elongated and round), and reconstructed their phylogenetic relationships with other acantharian sequences (Figure 2; Table S1). Photos of the cyst types and the most genetically related adult specimens (vegetative stage) are shown in Figure 2. The walls of all cysts showed numerous pore openings, but no small mineral plates were observed. All cysts sampled in this study belonged to the early diverging clades A, B and C, representing the taxonomic orders Chaunacanthida and Holacanthida. No cyst sequences were affiliated with clades E and F, which consist of taxa that consistently harbor symbiotic microalgae. The pear-shaped cyst (cyst 50), found at 620 meters depth, branched within clade A (previously named clade II in [15]), and was closely related to the genus *Acanthoplegma* sp. (Ei 59) in the order Holacanthida. All the elongated cysts (>10 specimens were sequenced), collected at different depths up to 2000 meters, are genetically identical and hold a phylogenetic position close to the specimen Ei 68 in subclade B1 (information about cysts are detailed in Table S1). The round cysts (cyst 25, 28, 45, 48) sampled at the same depths grouped within clade C (subclade C3), which represents the order Chaunacanthida. The numerous elongated and round cysts were observed through the microscope from the three different trap samples (160 m, 620 m and 2000 m). Each type of cyst exhibited some morphological variations according to the depth despite the same genetic identity: spicules were still visible at 160 m but appeared partly resorbed at greater depths, and were totally absent in cysts from 2000 m. Two other round cysts with large orifices at one pole (Vil 162 and Pec 9) were found at the surface in the Mediterranean Sea and belonged to subclade C4. The individual Vil 162 was particularly closely related to the adult species *Gigartacon muelleri* (Vil 105, 117, 41, 52, 53, and 61): the 18S rRNA and 28S rRNA gene sequences were 100% and 99% identical, respectively.

Several encystment events of the species *Acanthochiasma* sp. (Holacanthida) were observed and isolated through the microscope (Video S1). While crawling rapidly on the Petri dish, the cells expelled all their spicules but one, and excreted cytoplasmic remains and microalgal cells (presumably symbionts). Cyst formation was not completed in most cases because the cells died beforehand, but the final shape of the cyst that we could observe was round. These cysts (Pec 14, Pec 18, Vil 20) had a similar genetic identity and belonged to clade B2.

Vegetative cells were also found in trap samples at 160 m (adult 20) and 620 m (adult 43 and 52), and their sequences grouped within clade C3 and F, respectively.

### Biogeography and vertical distribution of cyst-forming Acantharia

#### Clone libraries

**Figure 3:**
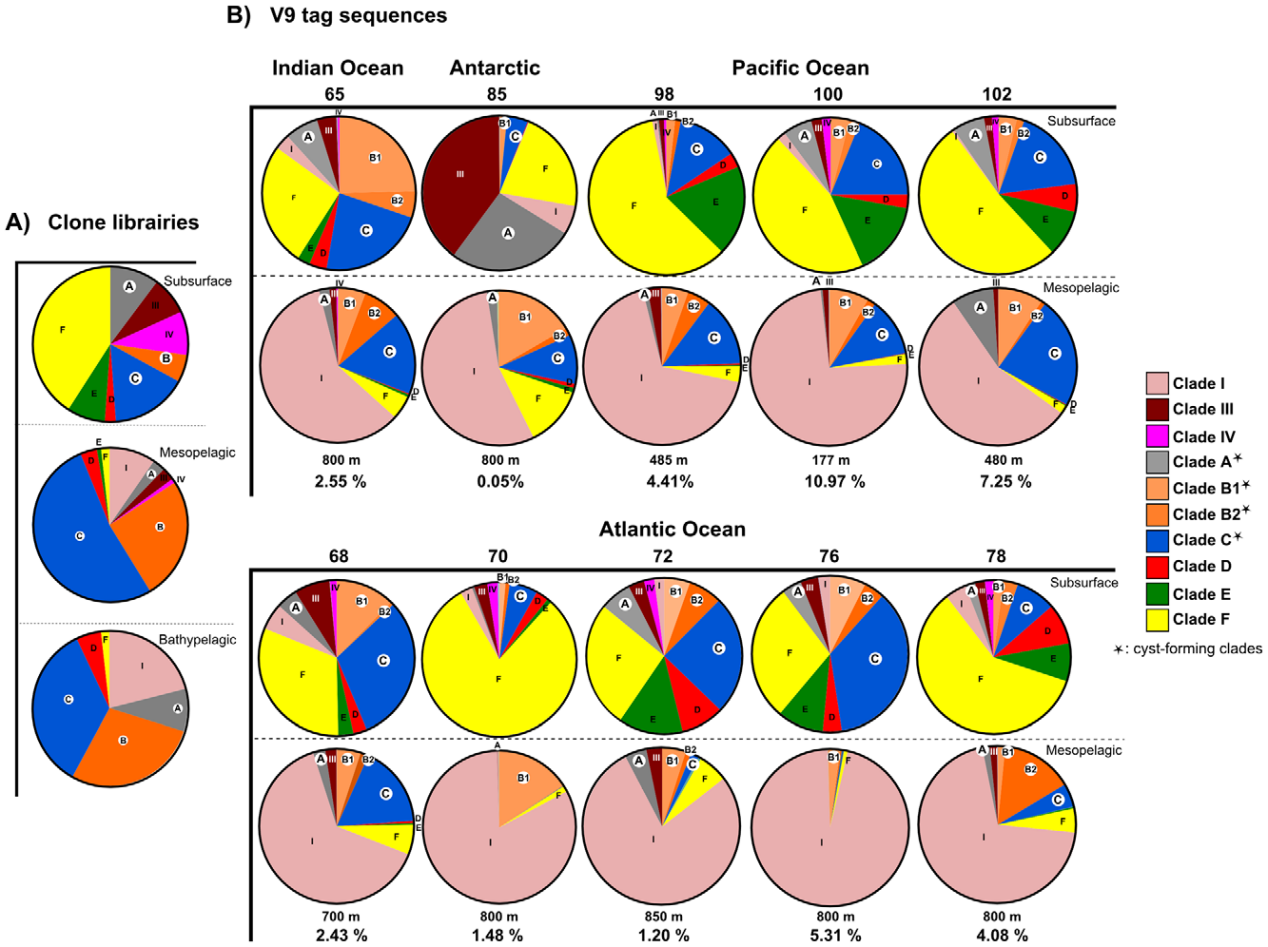
Vertical distribution of Acantharia based on 18S rRNA environmental sequences. A) clone libraries obtained from previous studies in different oceanic regions; B) V9 tag sequences from the present study sampled in the Indian (station 65), Antarctic (station 85), Pacific (stations 98 to 102) and Atlantic Oceans (stations 68 to 78) from different size fractions (0.8–5 µm and 20–2000 µm). In the pie-charts, cyst-forming clades A, B and C are highlighted with a white circle, and clades representing <1% of the total acantharian sequences are not shown. For the mesopelagic zone, the sampling depth and the contribution of acantharian sequences to total protistan sequences are indicated below the pie-charts.

We retrieved all the 18S rRNA gene sequences from GenBank related to Acantharia that were found in environmental clone libraries, and placed them with pplacer onto a reference phylogeny of Acantharia using the pplacer program (Figure S1). These environmental sequences were sampled between the surface and 3000 m in the Mediterranean Sea, and the Atlantic, Indian and Pacific Oceans, from different size fractions (total to <3 µm). We found 88 sequences between 0–100 m representing a wide genetic diversity of Acantharia (Figure 3A). Clades E and F accounted for about 50% of all 18S rRNA sequences, while the cyst forming ones accounted for 10%, 6% and 16% for A, B and C, respectively. Between 100–1000 m, which is essentially below the photic zone in most oceanic regions, clades B and C clearly dominated (more than 75% of the 114 acantharian sequences found at this depth), whereas the symbiotic clades E and F represented only 3% of sequences. Clade A contributed 3% between 100 and 1000 m, and clade I accounted for 10% in this zone. Clades I and III were named in a previous study [15] and are only represented by 18S rRNA environmental sequences (Figure S1). Between 1000 and 3000 m, the bathypelagic zone, clades B and C still represented a large proportion of acantharian sequences (60% out of 57 sequences), and clades I and A accounted for 21% and 9%, respectively. While no sequences of clades E were found in bathypelagic waters, clade F was represented by only one sequence in this zone. Thus, the cyst-forming clades A, B and C seemed to be present throughout the water column from the surface to 3000 m depth, and below the photic zone their sequences outnumbered those of other clades. Clade D was also present from the surface to the bathypelagic zone.

Many environmental sequences from clone libraries were very close genetically to the cyst sequences retrieved in this study. For the round and elongated cysts from clade C and B1, more than 50 associated sequences were found between 200 and 1000 meters, some from the anoxic Cariaco Basin, Venezuela [44]. The environmental sequences that were related to the cysts of clade B2 were sampled in the hydrothermal vent sediment of the Guaymas Basin [45], and in a low-oxygenated layer at 1000 m depth in the Marmara Sea [20]. Remarkably, the large majority of environmental sequences related to clades A, B and C, retrieved in mesopelagic and bathypelagic waters were found in the picoeukaryote size fraction (<3 µm). Clone libraries associated with Sanger sequencing generally represent the most abundant taxa in the environment because of the low sequencing depth (<1000 clones). Generating much more reads, the new high-throughput sequencing methods can allow us to have now a more comprehensive view of the protistan community.

#### High-throughput environmental V9 tag sequences

Environmental tags (V9 region of the 18S rRNA gene) were obtained at 10 locations in the South Indian, Atlantic and Pacific Oceans, in the photic and mesopelagic zones. The sampling depth in the mesopelagic zone varied between stations from 177 to 800 m (Table 1). In total, 236,666 and 755,538 V9 sequences assigned to different acantharian clades were retrieved from the surface and mesopelagic zone, respectively (Table S2), and found in both 0.8–5 µm and 20–2000 µm size fractions. As observed in the clone libraries, the acantharian community in the surface was generally greatly diversified with a higher number of sequences from the symbiotic clades E and F at most stations (Figure 3B). Yet, we can notice that at stations 65, 68, 72 and 76, cyst-forming clades A, B and C accounted for 44% to 60% of acantharian sequences. In the Southern Ocean (station 85), sampled in December, the surface community differed greatly from other stations with clades III and A representing 40% and 26%, respectively.

In the mesopelagic zone, the acantharian community was relatively similar throughout the Indian, Atlantic and Pacific Oceans, but systematically very different from that found at the surface. In these deep waters, acantharian sequences contributed between 4.4 and 11% in the Pacific and between 1.2 and 5.3% in the Atlantic of total protist sequences. Clade I represented 55 to 96% of the acantharian sequences in the mesopelagic, whereas it only accounted for ≤6% at the surface. In contrast, clades A, B and C were equally present at the surface and in the mesopelagic zone at most stations.

In order to gain a more accurate picture of the vertical distribution at the cyst-level, we focused on the environmental V9 sequences that were closely related to the cysts collected in this study (a threshold value of 97% was chosen). The end of the 18S rRNA gene (including the V9 region) was not PCR-amplified for the round (48, 45, 28 and 25) and pear-shaped cysts (cyst 50) because of the low PCR success for formaldehyde-preserved cysts. However, we obtained the V9 region for the cysts Vil 162, Pec 9, Cyst 6 and Vil 20 from subclades C4, B1 and B2, respectively. The vertical distribution of V9 sequences that shared more than 97% identity with the cyst sequences is shown in Table S3. While no V9 sequences related to Vil 162 were found, V9 sequences assigned to elongated cysts (cyst 6) were more numerous in the mesopelagic zone than in the photic zone where they were even absent in some cases (e.g. station 68). V9 sequences of Pec 9 and Vil 20 were either more abundant in the mesopelagic zone or in the photic layer depending on the station.

### Strontium and carbon fluxes of Acantharia across the Atlantic Ocean

**Figure 4:**
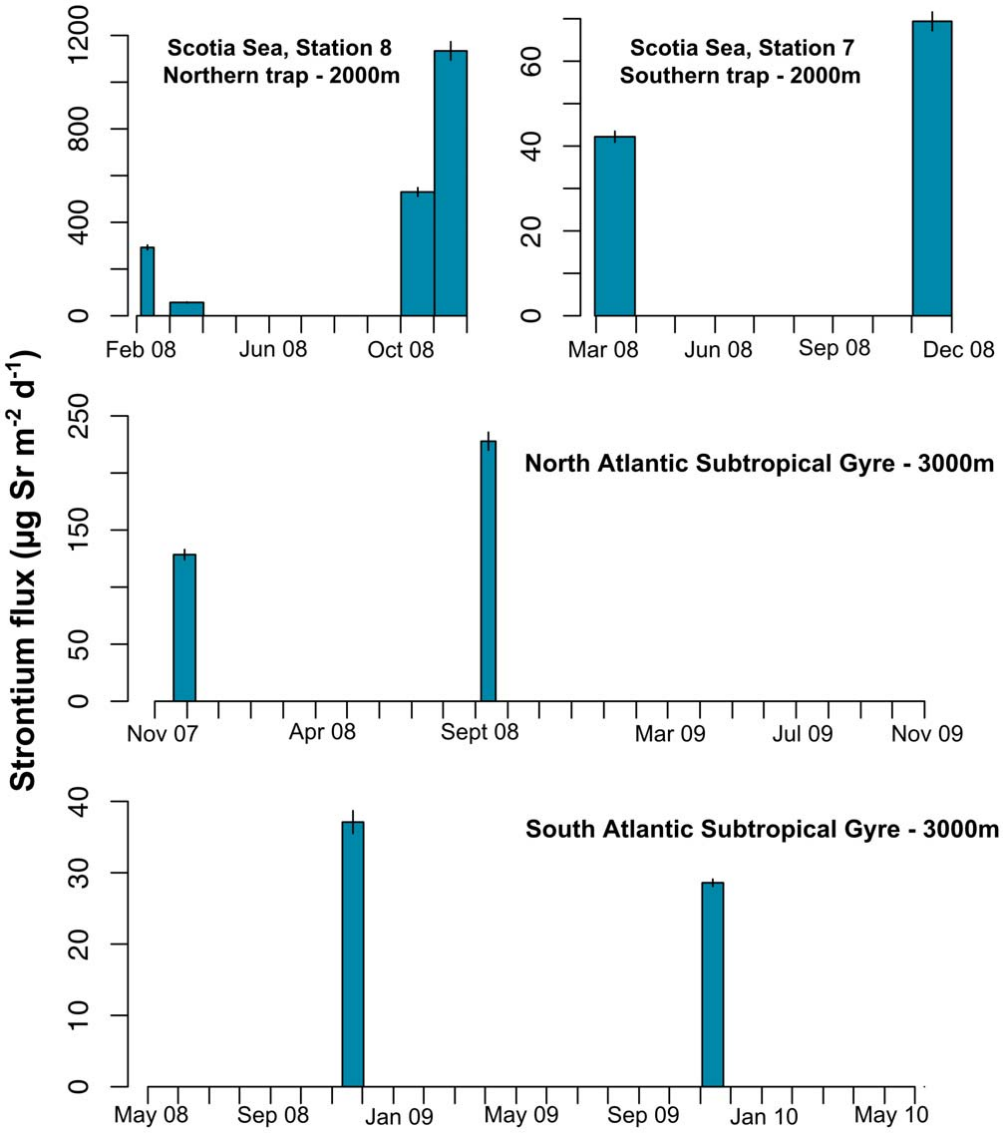
Geographic variation of the strontium flux. Strontium fluxes were measured in different sediment traps deployed across the Atlantic Ocean, in the Scotia Sea at 2000 m (St 7 and St 8; in 2008), and in the Northern and Southern subtropical gyres at 3000 m (2007–2010). Significant strontium fluxes are represented by a bar, from which the width is proportional to the collection period (around 14–31 days). Strontium concentrations for each sediment trap sample are shown in Figure S2.

We assessed the temporal pattern of acantharian vertical flux at different latitudes in the Atlantic Ocean with sediment traps in the Scotia Sea (2000 m) and the subtropical North and South Atlantic (NOG and SOG, 3000 m), deployed for one or two years. Sr fluxes were highly episodic, with no more than a third of all samples on a trap containing elevated Sr and never in more than two consecutive samples (Figures 4 and S2). Fluxes were very low in the subtropical traps, with only two samples in two separate years at each site containing significant fluxes: these were very low at the southern subtropical location (0.03 and 0.04 mg Sr m^−2^ d^−1^), and higher at the northern subtropical site (0.13 and 0.23 mg Sr m^−2^ d^−1^). Interestingly, these fluxes were all caught in boreal autumn: in November 2007 and September 2008 at the northern site, and in November 2008 and 2009 at the southern site, implying that there might be a seasonal cycle to acantharian sedimentation at both locations.

Fluxes in the Scotia Sea were similarly low at the southern site, with 0.02 and 0.04 mg Sr m^−2^ d^−1^ in March and November 2008. At the northern site, however, Sr fluxes of 0.03–0.64 mg Sr m^−2^ d^−1^ were caught in February, March, October, and November 2008.

We did not directly measure the acantharian POC fluxes. However, the C∶Sr ratio reported by Martin et al. [4] from the Iceland Basin at 2000 m (0.120±0.022 mg mg^−1^) would imply a contribution by Acantharia, during those collection periods in which Sr concentrations were elevated in the traps, of 0.004–0.003 mg C m^−2^ d^−1^ and 0.015–0.027 mg C m^−2^ d^−1^ at the southern and northern subtropical sites, respectively, and 0.005–0.008 mg C m^−2^ d^−1^ and 0.007–0.136 mg C m^−2^ d^−1^ at the southern and northern Scotia Sea site, respectively. Fluxes of total POC have not yet been measured from the subtropical traps, but are known for the Scotia Sea traps for each month (G. Tarling unpubl.). We hence estimate a contribution by Acantharia of 0.6–3.1% to the total monthly POC flux during the significant episodes of Sr flux at station 7 (March and November 2008) and at station 8 (February, March, October and November 2008) (Figures 4 and S2).

## Discussion

### Morphology of cysts and their vegetative stages

Encystment in Acantharia entails a remarkable morphological transformation whereby the diagnostic morphological features of the vegetative stage disappear. In this study, DNA identification allowed us to link cyst morphologies to vegetative cells of known taxonomy, and to highlight the cyst-forming acantharian clades. Cyst formation appears to be an old life-history trait in Acantharia since all cysts we sampled belonged to the early diverging clades A, B and C, representing the taxonomic orders Holacanthida and Chaunacanthida. Acantharia from these clades are characterized by identical long spicules that lack lateral extensions (apophyses) and are arranged by a loose central junction [15]. Some morphotypes of clade C (Chaunacanthida) can fold their spicules forming umbrella-like morphology (litholoph form), and the ones of clade B (Holacanthida) can totally shed their spicules. The cyst-forming Acantharia have thus a flexible mineral skeleton during their vegetative stage, and dissociation of the central junction seems to be a required stage for encystment. In contrast, non-cyst-forming individuals from other clades (IV, D, E and F), mainly the orders Symphiacanthida and Arthracanthida, have a fixed and unfolded skeleton because of a very tight central junction and robust spicules with apophyses. It has been observed that some Symphiacanthida (e.g. *Amphibelone* sp.) are also able to form cysts, whereby the cyst shell is deposited upon the skeleton of the vegetative cell [9], [14], but no molecular identification has confirmed the phylogenetic affiliation of these particular cysts. More cyst morphotypes have to be isolated and genetically identified to see whether other clades like I and III are also able to encyst and whether different encystment processes exist.

### Diversity of Acantharia in the mesopelagic and bathypelagic zones

Planktonic protists hold key roles in the trophic chain and metabolism of the mesopelagic and bathypelagic zones but, essentially due to sampling difficulties, little is known about their diversity in these deep waters [1]. Cloning and sequencing analyses suggest that the diversity of protists, as it is the case for bacteria [46], appears to decline in deep layers, where Radiolaria (including Acantharia), Alveolata and Euglenozoa have been reported to be the most abundant eukaryotic taxa [16], [18], [47]. Our study confirms that Acantharia, and more specifically clades I, A, B and C, can represent an important component of deep-sea protists worldwide, up to 11% of the environmental tag sequences. Yet, assuming that the number of rDNA copies scales with the number of nuclei, we cannot rule out that the multiple nuclei found in Acantharia might amplify their relative contribution in the microbial community [48]. But this bias is mitigated if one focuses specifically on the Acantharia, since all the species are polynucleated (except *Haliomatidium* sp., [49]).

Acantharia from clade I, the morphology of which is totally unknown, are predominantly found in the dark ocean (down to 3000 m) and constitute up to 75% of the acantharian community in the mesopelagic zone. This clade appeared to be specific, possibly endemic, to deep waters but we cannot say whether it can undertake encystment for reproduction. For clades A, B and C, the DNA identification of cysts leads us to suggest that their presence in deep waters is probably linked to their encystment capacity.

### Ecology and life cycle of cyst-forming Acantharia

The vertical distribution of the cyst-forming Acantharia revealed in this study can improve our understanding of the life cycle. The wide vertical distribution of clades A, B and C from the surface to the mesopelagic zone, detected by environmental clone libraries and V9 tag sequences (Figure 3), suggests that the vegetative stages occur in the upper layers and reproduction takes place in the dark ocean following encystment. Many sequences related to these clades were found in deep waters in the picoeukaryote size fraction (0.8–5 µm), much smaller than the size range of cysts (200–1000 µm). They rather correspond to the size range of acantharian swarmers (2–3 µm, [12]). Alternatively, one cannot rule out that a fraction of these DNA sequences would come from Acantharia-derived extracellular DNA or detritus produced by the different pumping steps during collection that end up in the smallest size fraction [16], [50]. While the ploidy of the swarmers is unknown, it is thought that they fuse and form juveniles, which then ascend into the upper layers [8]. Deep-sea bacterial and protistan communities are markedly different from the ones dwelling in the photic layer [17], [19] but this study shows that the life cycle of particular microorganisms can lead to their occurrence throughout the water column. DNA identification of two stages of life cycle, the vegetative and cyst stage here, can help interpreting the numerous forthcoming molecular surveys investigating the eukaryotic community in the photic and dark ocean [16], [19], [20], [51], [52], but also in sediment trap samples [53]. Ultimately, this highlights the significance of the life traits and ecology of the organisms for a better assessment of ocean biogeochemistry.

Remarkably, our phylogenetic analysis indicates that most cyst-forming Acantharia do not harbor symbiotic microalgae. The species *Acanthochiasma* sp. of clade B2 is an exception, since microalgae have been observed in symbiosis [54]. Symbiotic microalgae have otherwise only been found in the more recently diverging clades E and F (Arthracanthida and Symphiacanthida), in which sexual reproduction takes place directly from the vegetative stage without encystment [8]. This obligate and horizontally transmitted symbiosis would constrain the hosts to remain and complete their life cycle in the photic zone for rapid acquisition and maintenance of their photosynthetic symbionts. This is consistent with the very few sequences from clades E and F being found in the mesopelagic and below in most oceanic regions (Figure 3). In contrast, non-symbiotic Acantharia could theoretically occupy any depth in the water column provided that food availability was sufficient. Besides, in contrast to the orders Arthracanthida and Symphiacanthida, the Holacanthida and Chaunacanthida have a less developed buoyancy apparatus with few myonemes (non-actin filaments) per spicules [8], [55], suggesting that fine buoyancy control might be less critical for cyst-forming Acantharia. Of course, at this status of knowledge, we cannot rule out the possibility that acantharian species within a single clade have different niches in the water column.

**Figure 5:**
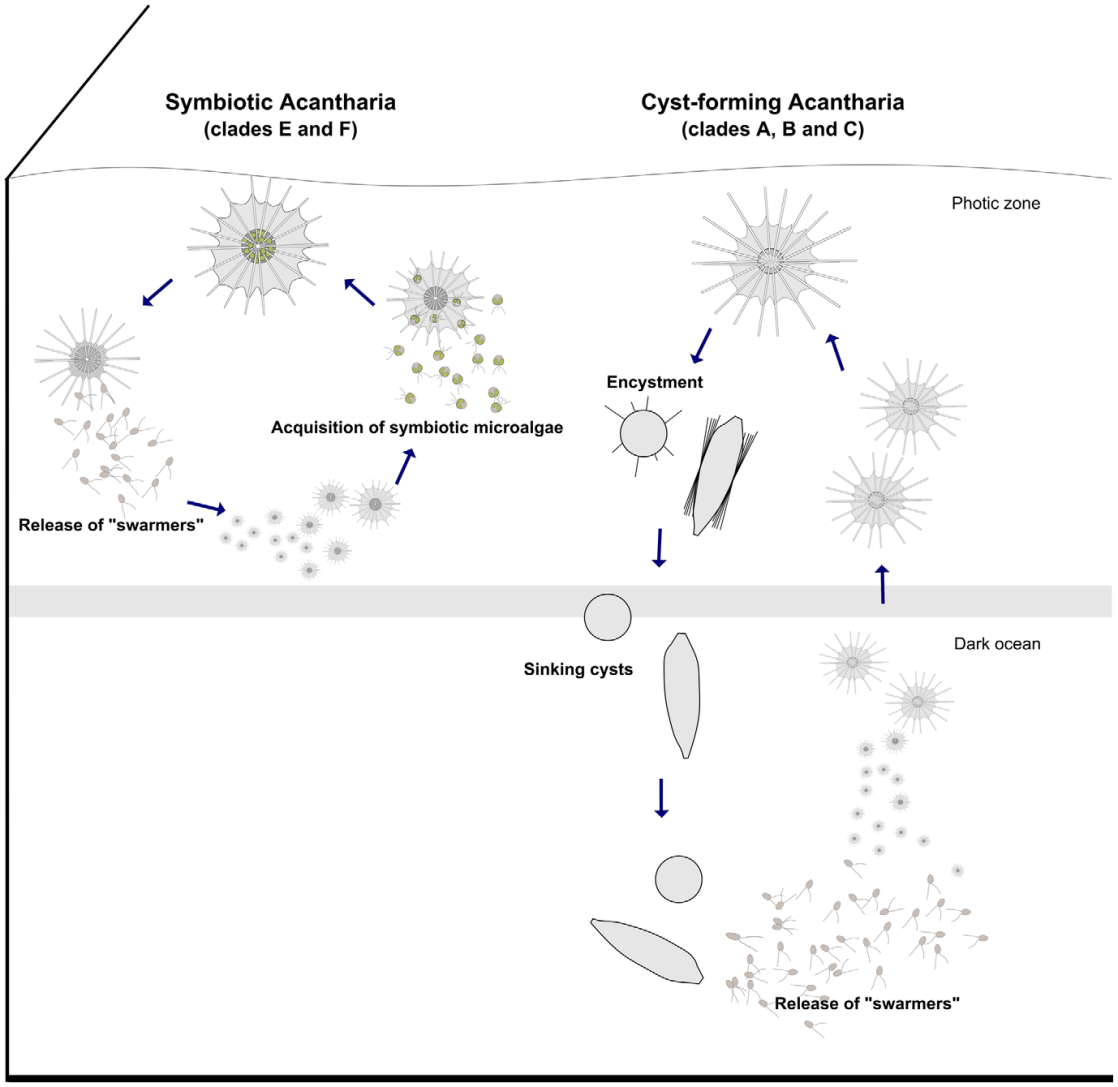
Hypothetical scenario of the life cycle in symbiotic and cyst-forming Acantharia with shallow and deep reproduction, respectively.

Distinct shallow and deep modes of reproduction have been also reported in planktonic Foraminifera, a sister group of the Radiolaria [56], [57]. Likewise, foraminiferan species that have part of their life cycle below the photic layer, are not associated with symbiotic microalgae, indicating that photosymbiosis strongly influences the life cycle and consequently the vertical distribution of hosts. A hypothetical scenario for the life cycle of cyst-forming and symbiotic Acantharia is proposed in Figure 5. Our study suggests that cyst-forming Acantharia do not contribute to primary production in the photic layer, a biogeochemical role specifically attributed to symbiotic forms [58], but do participate in the vertical particle flux in the oceans.

### Geographic variation of the acantharian contribution to downward particle flux

Mineralized planktonic organisms, such as Foraminifera, coccolithophorids, diatoms and Radiolaria, are important contributors to downward particle flux in the ocean and thus play key roles in global element cycles [2], [59], [60]. Encystment in Acantharia is another example where changes in the life cycle of upper ocean organisms have a direct effect on downward particle flux. Acantharian fluxes in the bathypelagic were very low in the subtropical Atlantic, but higher in the Scotia Sea. Together with the data presented previously [4], [31], our results support the conclusion that Acantharia only sink past the upper mesopelagic in appreciable numbers at high latitude. Our estimates of acantharian POC fluxes at the northern Scotia Sea site are some of the highest reported from the bathypelagic, even though the percentage contribution to total POC flux was minor. In the Iceland Basin, acantharian cyst flux could contribute up to 48% and 59% of total bathypelagic POC and particulate nitrogen flux during the two weeks before spring bloom phytodetritus was caught in the sediment traps [4]. In the East Greenland Sea, acantharian cysts comprised up to 90% of the POC sinking at 100 m during a short-term sampling period [21]. Thus, Acantharia can contribute to long-term carbon sequestration in the deep-sea. In subtropical regions, mainly oligotrophic, the vertical flux of acantharian cyst is lower and explains why modest values were detected at 3000 m depth in this study. In the Mediterranean Sea and Indian Ocean, cysts were generally reported only between 50 and 250 m [9], [11].

Since cysts are more heavily mineralized and thus less susceptible to dissolution than vegetative cells, we suspect that cysts would have contributed the majority of the fluxes in our traps. Nevertheless, a part of the strontium measured in sediment traps can still originate from skeletons of deep-dwelling species. Swarmers of colonial Radiolaria (Collodaria) are also known to contain small crystals of celestite [61]. However, Collodaria typically dwell in the upper layer of the photic zone [62] and accomplish their reproduction without encystment. Since swarmers are in the low micrometer size-range, they would have very low sinking speeds regardless of ballast mineral content. Hence it is unlikely that they would contribute substantially to mesopelagic and bathypelagic strontium flux compared to acantharian cysts.

We do not believe that artefacts may have significantly biased our regional sediment trap comparison. We can rule out a significant contribution of Sr by CaCO_3_ dissolution, as Ca was never elevated by more than 300 µg mL^−1^ above samples that contained only background Sr concentrations. Since CaCO_3_ typically contains no more than ∼1 mg Sr g^−1^, CaCO_3_ is unlikely to have contributed more than 0.3 µg Sr mL^−1^. While the Sr fluxes we report from the northern Scotia Sea are still lower than those reported from the Iceland Basin [4], they are nevertheless comparable to fluxes reported elsewhere above 500 m [3], [26], [63].

The difference in vertical flux of Acantharia between low and high latitudes is potentially due to larger cysts with higher sinking rates being found in high latitudes. Cysts of clade B1 collected in the Iceland Basin were up to 1 mm long and had sank of 490±150 m d^−1^ [4], while cysts reported by Bernstein et al. [3] in the North Pacific Ocean were only a few hundred µm in diameter. Yet, cysts morphologically similar to the Iceland Basin cysts (clade B1) have been observed not only in the Southern [64] but also in the Indian Ocean [11] and the South China Sea [14], indicating that large cysts may not necessarily be restricted to cold waters in high-latitudes. Moreover, Antia et al. [21] reported small cysts with low sinking rates from the Greenland Sea, one of the highest latitude sites sampled for Acantharia.

A different possibility is that cyst-forming Acantharia in general might be more abundant at higher latitudes. We found that cyst-forming clades are widely distributed in surface waters of the Indian, Atlantic and Pacific Oceans but tend to be more prominent at the surface of nutrient-rich coastal areas (Figure 3): clades B and C in stations 65, 68 off South Africa, and clade A in Southern Ocean (station 85). It is interesting to note that station 85 was sampled in austral summer (December), a period matching with a significant strontium flux in the Scotia Sea (Figures 4 and S2). In addition, Popofsky [10] observed that the species *Acanthochiasma* sp. (a species found in clade B) becomes abundant during the summer in the Antarctic. In contrast, clades E and F that do not encyst are predominant in the oligotrophic open ocean. Higher latitudes might thus be home to larger numbers of cyst-forming species at a given time than subtropical regions, giving rise to higher downward fluxes of acantharian cysts. However, it is difficult to compare accurately the acantharian communities found in different oceanic regions because we could not address temporal variation at each sampling location.

### Seasonal variation of acantharian cyst sedimentation

Long-term sediment trap deployments across a latitudinal gradient allowed us to follow the seasonal dynamics of the acantharian sinking flux. Strontium fluxes in both subtropical and Scotia Sea traps showed a marked seasonal pattern, as observed in the Southern Ocean and Iceland Basin, where cysts were only caught in summer (Scotia Sea) and spring (Iceland Basin), respectively [4], [64]. For traps deployed in the Scotia Sea, the months December and January were not sampled, though it is possible that strontium flux was significant at this time, given the temporal pattern (Figures 4 and S2). Cyst formation and sedimentation are hence presumably driven by environmental cues. The strong difference in Sr flux between the two Scotia Sea sites suggests that acantharian fluxes are indeed linked to phytoplankton blooms, as hypothesized by Martin et al. [4]: the northern Scotia Sea site experienced intense phytoplankton blooms starting in October/November, and the northern sediment trap showed peaks in particulate carbon flux in October and November 2008, rising from around 50 mg C m^−2^ d^−1^ in September to >400 mg C m^−2^ d^−1^ in October and November [65]. The southern trap shows no such sharp peak, and instead had the highest carbon fluxes in March and May. Thus, formation and deep sinking of cysts leading to reproduction of these acantharian clades does appear to be linked to primary productivity. This mirrors the periodicity of encystment in marine ciliates in summer [66], and that in a freshwater ciliate in May and September [67], which are both related to chlorophyll-*a* concentration. We therefore suggest that cyst-forming Acantharian show a clear seasonality in high-latitude waters that tracks phytoplankton stocks, and then complete their life cycle by sinking deep into the interior of the ocean.

### Hypotheses on the ecological role of encystment in Acantharia

Encystment in Acantharia is not a reversible resting stage like in many planktonic protists, but rather a key step for reproduction. Cyst formation is clearly an active process, and evidently an efficient mechanism for rapid sinking. While we do not know anything specific about the bioenergetics of celestite precipitation, it is possible that the extensive biomineralisation and remodelling of the cyst shell is not a trivial energy expense. To our knowledge, such a reproductive strategy has not been reported for other marine protists. The robust seed-like shells not only act as ballast but might also protect cysts from grazing during descent. Although it is difficult to assert the true ecological role of this life-history trait, sinking in the water column for reproduction must have an adaptive value for heterotrophic protists. In stratified waters, rapid sinking might allow individuals to quickly reach depths at which predation risk is reduced. In phytoplankton, vertical migration is a common strategy to increase nutrient uptake when surface waters are nutrient-depleted (e.g. the diatom *Rhizosolenia* sp., [68], [69]). However, for heterotrophs like cyst-forming Acantharia, sinking into the dark ocean where food concentrations are lower than in surface waters may entail trade-offs. While we know little about their diet, Acantharia are known to ingest a wide range of prey, such as bacteria, microalgae and even zooplankton [70]. It is interesting to note in this context that Schewiakoff ([8] p. 686) reported that 73% of Acantharia in which he could see food vacuoles were caught below 100 m depth. We hence suggest that cyst-forming Acantharia at high latitudes and during phytoplankton blooms, when downward particle flux shows sudden spikes, sink particularly deep because juveniles could still exploit the fresh phytodetritus that sinks right to the seafloor at this time. Conversely, in low-latitude settings in which production and downward particle flux are far lower and more constant, sinking very deep may be less advantageous as far less fresh particulate matter sinks to great depths here. The regional and temporal flux data we have presented here are consistent with this view.

## Supporting Information

Figure S1 **Diversity of acantharian sequences found in deep waters.** Pplacer tree showing the phylogenetic placement of 260 environmental partial 18S rRNA sequences sampled in previous studies into reference clades of Acantharia (I, III, IV, A–F). GenBank accession numbers of these environmental sequences are indicated in the taxon name. The depth at which the acantharian sequences were collected is indicated with the blue bars (the length of the bar scales with the sampling depth of the environmental sequence).
(TIF)

Figure S2 **Temporal pattern of strontium flux across the Atlantic Ocean.** Concentration of dissolved strontium measured in each sample cup of the four bathypelagic sediment traps, which were deployed at 2000 m in the Scotia Sea (St 7 and St 8; 2008) and at 3000 m in the Northern and Southern subtropical gyres (2007–2010). The horizontal dashed line represents the background strontium concentration above which the Sr flux from Acantharia was deemed significant.
(TIF)

Table S1 Information about the cysts collected in this study and the acantharian species used for the molecular phylogeny. Species and order names, and GenBank accession numbers of the 18S and 28S rRNA gene are given. Code names in bold indicate that the sample have been collected in a sediment trap.
(PDF)

Table S2 Number of V9 tag sequences of the 18S rRNA, assigned to Acantharia and protists, at different stations in big (20–2000 µm) and small (0.8–5 µm) size fraction.
(PDF)

Table S3 Comparison of the number of V9 tag sequences assigned to different cysts (>97%) isolated in this study between the photic and the mesopelagic zones.
(PDF)

Video S1 **Cyst formation observed through the microscope of an acantharian cell from clade B2.**
(WMV)

## References

[1] Aristegui J, Gasol JM, Duarte CM, Herndl GJ (2009) Microbial oceanography of the dark ocean's pelagic realm. Limnol Oceanogr 54 (5) 1501–1529.

[2] Lampitt RS, Salter I, John D (2009) Radiolaria: major exporters of organic carbon to the deep ocean. Global Biogeochem Cycles 23: GB1010 doi:10.1029/2008GB003221.

[3] Bernstein RE, Feely RA, Byrne RH, Lamb MF, Michaels AF (1987) Acantharian fluxes and strontium to chlorinity ratios in the north Pacific Ocean. Science 237: 1490–1494. 17816791 10.1126/science.237.4821.1490

[4] Martin P, Allen JT, Cooper MJ, Johns DG, Lampitt RS, et al (2010) Sedimentation of acantharian cysts in the Iceland Basin: Strontium as a ballast for deep ocean particle flux, and implications for acantharian reproductive strategies. Limnol Oceanogr 55: 604–614.

[5] Smetacek VS (1985) Role of sinking in diatom life-history cycles: ecological, evolutionary and geological significance. Mar Biol 84: 239–251.

[6] Kremp A, Rengefors F, Montresor M (2009) Species specific encystment patterns in three Baltic cold-water dinoflagellates: the role of multiple cues in resting cyst formation. Limnol Oceanogr 54 (4) 1125–1138.

[7] Von Dassow P, Montresor M (2011) Unveiling the mysteries of phytoplankton life cycles: patterns and opportunities behind complexity. J Plankton Res 33 (1) 3–12.

[8] Schewiakoff WT (1926) Die Acantharia. Fauna e Flora del Golfo di Napoli 37: 1–755.

[9] Hollande A, Cachon J, Cachon-Enjumet M (1965) Les modalités de l'enkystement présporogénétique chez les acanthaires. Protistologica 1: 91–112 [In French].

[10] Popofsky A (1908) Die Radiolarien der Antarktis (mit Ausnahme der Tripyleen) Deutsche Südpolar-Expedition 1901–1903,. Zoologie II 10 (3) 183–305 [In German].

[11] Bottazzi EM (1973) Ulteriori ritrovamenti di cisti di Acantari (protozoa). Mt Lomb Sci Lett 107B: 3–26.

[12] Decelle J, Probert I, Bittner L, Desdevises Y, Colin S, et al (2012) An original mode of symbiosis in open ocean plankton. Proc Natl Acad Sci USA 109 (44) 18000–18005. 23071304 10.1073/pnas.1212303109 PMC3497740

[13] Haeckel E (1888) Die Radiolarien (Rhizopoda Radiolaria). Eine Monographie, Dritter Teil, Die Acantharien oder Actinopyleen-Radiolarien. Verlag von Georg Reimer, Berlin, pp 33.

[14] Zhiyuan T, Xinghui S (1989) Studies on the acantharian cysts of the South China Sea? Studia Marina Sinica 30: 127–142.

[15] Decelle J, Suzuki N, Mahé F, de Vargas C, Not F (2012) Molecular phylogeny and morphological evolution of the Acantharia (Radiolaria). Protist 163: 435–450. 22154393 10.1016/j.protis.2011.10.002

[16] Gilg IC, Amaral-Zettler LA, Countway PD, Moorthi S, Schnetzer A, et al (2010) Phylogenetic affiliations of mesopelagic acantharia and acantharian-like environmental 18S rRNA genes off the Southern California coast. Protist 161: 197–211. 20044311 10.1016/j.protis.2009.09.002

[17] Not F, Gausling R, Azam F, Heidelberg JF, Worden AZ (2007) Vertical distribution of picoeukaryotic diversity in the Sargasso Sea. Environ Microbiol 9 (5) 1233–1252. 17472637 10.1111/j.1462-2920.2007.01247.x

[18] Schnetzer A, Moorthi SD, Countway PD, Gast RJ, Gilg IC, et al (2011) Depth matters: Microbial eukaryote diversity and community structure in the eastern North Pacific revealed through environmental gene libraries. Deep-Sea Res I 58: 16–26.

[19] Countway PD, Caron DA, Gast RJ, Savai P (2007) Comparison of protistan diversity in deep (2500 m) vs euphotic zone assemblages in the Sargasso Sea and Gulf Stream (N.Atlantic). Environ Microbiol 9: 1219–1232. 17472636 10.1111/j.1462-2920.2007.01243.x

[20] Quaiser A, Zivanovic Y, Moreira D, López-García P (2010) Comparative metagenomics of bathypelagic plankton and bottom sediment from the Sea of Marmara. ISME J 5: 285–304. 20668488 10.1038/ismej.2010.113 PMC3105693

[21] Antia AN, Bauerfeind E, Bodungen BV, Zeller U (1993) Abundance, encystment and sedimentation of acantharia during autumn 1990 in the East Greenland Sea. J Plankton Res 15: 99–114.

[22] Hollande A, Cachon-Enjumet M (1957) Enkystement et reproduction isosporogénétique chez les acanthaires. CR Acad Sci 244: 508–510 [In French].

[23] Kwon EY, Primeau F, Sarmiento JL (2009) The impact of remineralization depth on the air-sea carbon balance. Nat Geosci 2: 630–625.

[24] Beers JR, Stewart GL (1970) The preservation of acantharians in fixed plankton samples. Limnol Oceanogr 15: 825–827.

[25] Michaels AF, Caron DA, Swanberg NR, Howse FA, Michaels CM (1995) Planktonic sarcodines (Acantharia, Radiolaria, Foraminifera) in surface waters near Bermuda – abundance, biomass and vertical flux. J Plankton Res 17: 131–163.

[26] Bernstein RE, Byrne RH, Betzer PR, Greco AM (1992) Morphologies and transformations of celestite in seawater: the role of acantharians in strontium and barium geochemistry. Geochim Cosmochim Acta 56: 3273–3279.

[27] De Villiers S (1999) Seawater strontium and Sr/Ca variability in the Atlantic and Pacific oceans. Earth Planet Sci Lett 171: 623–634.

[28] De Dekker P (2004) On the celestite-secreting Acantharia and their effect on seawater strontium to calcium ratios. Hydrobiologia 517: 1–13.

[29] Bidwell JP, Paige JA, Kuzirian AM (1986) Effects of strontium on the embryonic development of *Aplysia californica* . Biological Bulletin (Woods Hole, MA, United States) 170 (1) 75–901.

[30] Hanlon RT, Bidwell JP, Tait R (1989) Strontium is required for statolith development and thus normal swimming behavior of hatchling cephalopods. J Exp Biol 141: 187–95. 2926318 10.1242/jeb.141.1.187

[31] Martin P, Lampitt RS, Perry MJ, Sanders R, Lee C, et al (2011) Export and mesopelagic particle flux during a North Atlantic spring diatom bloom. Deep-Sea Res I 58: 338–349 doi:10.1016/j.dsr.2011.01.006.

[32] Gouy M, Guindon S, Gascuel O (2010) SeaView version 4: a multiplatform graphical user interface for sequence alignment and phylogenetic tree building. Mol Biol Evol 27 (2) 221–224. 19854763 10.1093/molbev/msp259

[33] Vaidya G, Lohman DJ, Meier R (in press) SequenceMatrix: concatenation software for the fast assembly of multigene datasets with character set and codon information. Cladistics 10.1111/j.1096-0031.2010.00329.x 34875773

[34] Tamura K, Peterson D, Peterson N, Stecher G, Nei M, et al (2011) MEGA5: Molecular Evolutionary Genetics Analysis using Maximum Likelihood, Evolutionary Distance, and Maximum Parsimony Methods. Mol Biol Evol 28: 2731–2739. 21546353 10.1093/molbev/msr121 PMC3203626

[35] Guindon S, Gascuel O (2003) A simple, fast, and accurate algorithm to estimate large phylogenies by maximum likelihood. Syst Biol 52: 694–704. 10.1080/10635150390235520 14530136

[36] Drummond AJ, Rambaut A (2007) BEAST: Bayesian evolutionary analysis by sampling trees. BMC Evol Biol 7: 214. 17996036 10.1186/1471-2148-7-214 PMC2247476

[37] Matsen FA, Kodner RB, Armbrust EV (2010) pplacer: linear time maximum-likelihood and Bayesian phylogenetic placement of sequences onto a fixed reference tree. BMC Bioinformatics 11: 538. 21034504 10.1186/1471-2105-11-538 PMC3098090

[38] Karsenti E, Acinas SG, Bork P, Bowler C, De Vargas C, et al (2011) A Holistic Approach to Marine Eco-Systems Biology. PLoS Biol 9 (10) e1001177 doi:10.1371/journal.pbio.1001177. 22028628 10.1371/journal.pbio.1001177 PMC3196472

[39] Amaral-Zettler LA, McCliment EA, Ducklow HW, Huse SM (2009) A method for studying protistan diversity using massively parallel sequencing of V9 hypervariable regions of small-subunit ribosomal RNA genes. PLoS ONE 4 (7) e6372 doi:10.1371/journal.pone.0006372. 19633714 10.1371/journal.pone.0006372 PMC2711349

[40] Edgar RC (2010) Search and clustering orders of magnitude faster than BLAST. Bioinformatics 26 (19) 2460–2461. 20709691 10.1093/bioinformatics/btq461

[41] Guillou L, Bachar D, Audic S, Bass D, Berney C, et al (2012) The Protist Ribosomal Reference database (PR^2^): a catalog of unicellular eukaryote Small SubUnit rRNA sequences with curated taxonomy. Nucleic Acids Res 1–8 10.1093/nar/gks1160. 10.1093/nar/gks1160 PMC3531120 23193267

[42] Needleman SB, Wunsch CD (1970) A general method applicable to the search for similarities in the amino acid sequence of two proteins. J Mol Biol 48 (3) 443–453. 5420325 10.1016/0022-2836(70)90057-4

[43] Pearson WR, Lipman DJ (1988) Improved tools for biological sequence comparison. Proc Natl Acad Sci USA 85: 2444–2448. 3162770 10.1073/pnas.85.8.2444 PMC280013

[44] Edgcomb VP, Orsi W, Bunge J, Jeon S, Christen R, et al (2011) Protistan microbial observatory in the Cariaco Basin, Caribbean. I. Pyrosequencing vs Sanger insights into species richness. ISME J 5: 1344–1356. 21390079 10.1038/ismej.2011.6 PMC3146274

[45] Edgcomb VP, Kysela DT, Teske A, Gomez AD, Sogin ML (2002) Benthic eukaryotic diversity in the Guaymas Basin hydrothermal vent environment. Proc Natl Acad Sci USA 99: 7658–7662. 12032339 10.1073/pnas.062186399 PMC124314

[46] Hewson I, Steele JA, Capone DG, Fuhrman JA (2006) Remarkable heterogeneity in meso- and bathypelagic bacterioplankton assemblage composition. Limnol Oceanogr 51: 1274–1283.

[47] Shah Salani F, Arndt H, Hausmann K, Nitsche F, Scheckenbach F (2012) Analysis of the community structure of abyssal kinetoplastids revealed similar communities at larger spatial scales. ISME J 6: 713–723. 22071346 10.1038/ismej.2011.138 PMC3309350

[48] Suzuki N, Ogane K, Aita Y, Kato M, Sakai S, et al (2009) Distribution Patterns of the Radiolarian Nuclei and Symbionts Using DAPI-Fluorescence. Bull Natl Mus Nat Sci, Ser B 35: 169–182.

[49] Febvre J, Febvre C, Michaels A (2000) Acantharia Haeckel. (1881). In Lee JJ, Leedale GF, Bradbury P (Eds) An Illustrated Guide to the Protozoa (2nd edition). Organisms Traditionally Referred to as Protozoa, or Newly Discovered Groups, Society of Protozoologists, Kansas, 783–803.

[50] Not F, del Campo J, Balagué V, de Vargas C, Massana R (2009) New Insights into the Diversity of Marine Picoeukaryotes. PLoS ONE 4 (9) e7143 doi:10.1371/journal.pone.0007143. 19787059 10.1371/journal.pone.0007143 PMC2747013

[51] López-García P, Rodríguez-Valera F, Pedrós-Alió C, Moreira D (2001) Unexpected diversity of small eukaryotes in deep-sea Antarctic plankton. Nature 409: 603–607. 11214316 10.1038/35054537

[52] López-García P, Philippe H, Gail F, Moreira D (2003) Autochthonous eukaryotic diversity in hydrothermal sediment and experimental microcolonizers at the mid-Atlantic ridge. Proc Natl Acad Sci USA 100: 697–702. 12522264 10.1073/pnas.0235779100 PMC141059

[53] Amacher J, Neuer S, Anderson I, Massana R (2009) Molecular approach to determine contributions of the protist community to particle flux. Deep-Sea Res I 56: 2206–2215.

[54] Decelle J, Siano R, Probert I, Poirier C, Not F (2012) Multiple microalgal partners in symbiosis with the acantharian *Acanthochiasma* sp. (Radiolaria). Symbiosis doi:10.1007/s13199-012-0195-x.

[55] Febvre J, Febvre-Chevalier C (2001) Acantharia. In: eLS. John Wiley & Sons Ltd, Chichester. Available: http://www.els.net. doi: 10.1038/npg.els.0002102.

[56] Schiebel R, Hemleben C (2005) Modern planktic foraminifera. Paläontologische Zeitschrift 79: 135–148.

[57] Shaked Y, de Vargas C (2006) Pelagic photosymbiosis: rDNA assessment of diversity and evolution of dinoflagellate symbionts and planktonic foraminiferal hosts. Mar Ecol Prog Ser 325: 59–71.

[58] Michaels AF (1988) Vertical distribution and abundance of Acantharia and their symbionts. Mar Biol 97: 559–569.

[59] Salter I, Kemp AES, Moore CM, Lampitt RS, Wolff GA, et al (2012) Diatom resting spore ecology drives enhanced carbon export from a naturally iron-fertilized bloom in the Southern Ocean. Global Biogeochem Cycles 26: GB1014 doi:10.1029/2010GB003977.

[60] Schiebel R (2002) Planktic foraminiferal sedimentation and the marine calcite budget. Global Biogeochem Cycles 16: 1065.

[61] Anderson OR, Perry C, Hughes N (1990) Transmission and scanning electron microscopic evidence for cytoplasmic deposition of strontium sulphate crystals in colonial Radiolaria. Philosophical Transactions of the Royal Society of London B Biological Sciences 329: 81–86.

[62] Dennett MR, Caron DA, Michaels AF, Gallager SM, Davis CS (2002) Video plankton recorder reveals high abundances of colonial Radiolaria in surface waters of the central North Pacific. J Plankton Res 24: 797–805.

[63] Lamborg CH, Buesseler KO, Valdes J, Bertrand CH, Bidigare R, et al (2008) The flux of bio- and lithogenic material associated with sinking particles in the mesopelagic “twilight zone” of the northwest and North Central Pacific Ocean. Deep-Sea Res II 55: 1540–1563.

[64] Spindler M, Beyer K (1990) Distribution, abundance and diversity of Antarctic acantharian cysts. Mar Micropal 15: 209–218.

[65] Whitehouse MJ, Atkinson A, Korb RE, Venables HJ, Pond DW, et al (2012) Substantial primary production in the land-remote region of the central and northern Scotia Sea. Deep-Sea Res II 59–60: 47–56.

[66] Reid PC (1987) Mass encystment of a planktonic oligotrich ciliate. Mar Biol 95: 221–230.

[67] Müller H, Wünsch C (1999) Seasonal dynamics of cyst formation of pelagic strombidiid ciliates in a deep prealpine lake. Aquat Microb Ecol 17: 37–47.

[68] Richardson TL, Cullen JJ, Kelley DE, Lewis MR (1998) Potential contributions of vertically migrating *Rhizosolenia* to nutrient cycling and new production in the open ocean. J Plankton Res 20 (2) 219–241.

[69] Villareal TA, Altabet MA, Culver-Rymsza K (1993) Nitrogen transport by vertically migrating diatom mats in the North Pacific Ocean. Nature 363: 709–712.

[70] Swanberg NR, Caron DA (1991) Patterns of sarcodine feeding in epipelagic oceanic plankton. J Plankton Res 13 (2) 287–312.

