# Supplementary figures and images for: Diversity, Ecology and Biogeochemistry of Cyst-Forming Acantharia (Radiolaria) in the Oceans
Source: PLoS One. 2013 Jan 11;8(1):e53598. doi: 10.1371/journal.pone.0053598 (PMC3543462; doi:10.1371/journal.pone.0053598)

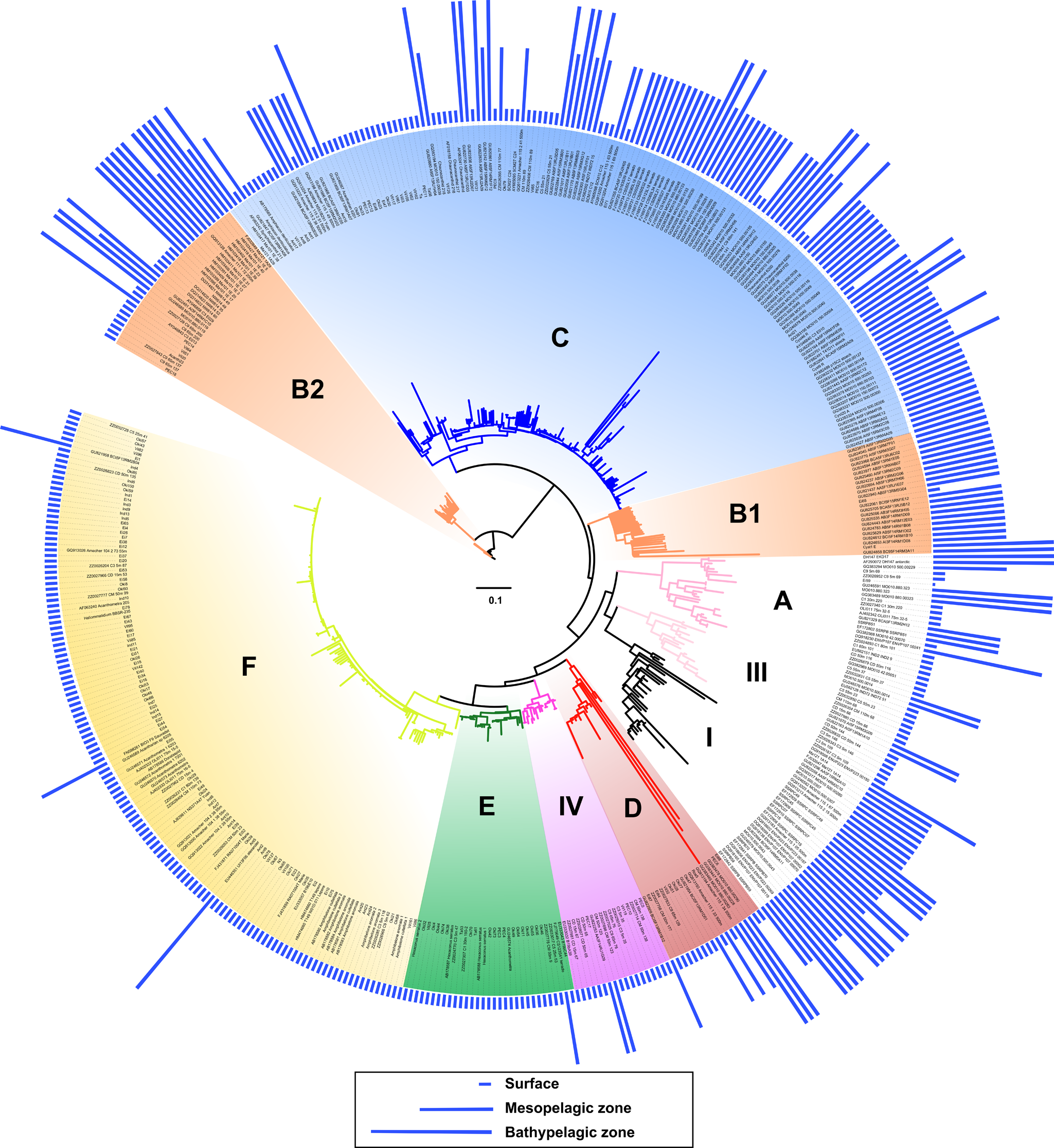

Supplement: Figure S1 — Diversity of acantharian sequences found in deep waters. Pplacer tree showing the phylogenetic placement of 260 environmental partial 18S rRNA sequences sampled in previous studies into reference clades of Acantharia (I, III, IV, A–F). GenBank accession numbers of these environmental sequences are indicated in the taxon name. The depth at which the acantharian sequences were collected is indicated with the blue bars (the length of the bar scales with the sampling depth of the environmental sequence). (TIF) [file pone.0053598.s001.tif]

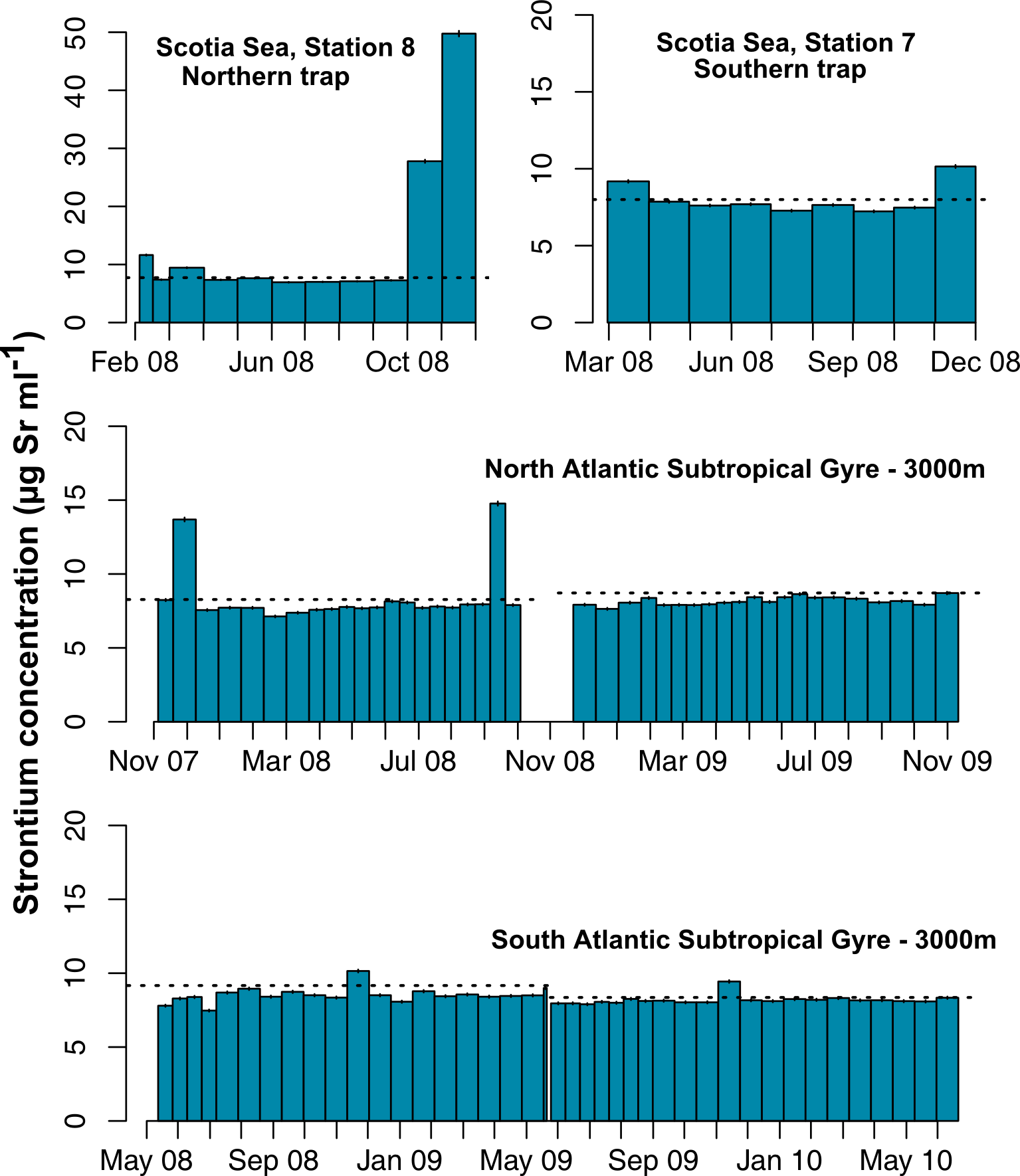

Supplement: Figure S2 — Temporal pattern of strontium flux across the Atlantic Ocean. Concentration of dissolved strontium measured in each sample cup of the four bathypelagic sediment traps, which were deployed at 2000 m in the Scotia Sea (St 7 and St 8; 2008) and at 3000 m in the Northern and Southern subtropical gyres (2007–2010). The horizontal dashed line represents the background strontium concentration above which the Sr flux from Acantharia was deemed significant. (TIF) [file pone.0053598.s002.tif]
