# Supplementary material for: Diversity, Ecology and Biogeochemistry of Cyst-Forming Acantharia (Radiolaria) in the Oceans
Source: PLoS One. 2013 Jan 11;8(1):e53598. doi: 10.1371/journal.pone.0053598 (PMC3543462; doi:10.1371/journal.pone.0053598)
Supplement: Table S1 — Information about the cysts collected in this study and the acantharian species used for the molecular phylogeny. Species and order names, and GenBank accession numbers of the 18S and 28S rRNA gene are given. Code names in bold indicate that the sample have been collected in a sediment trap. (PDF) [file pone.0053598.s003.pdf]

Code name	Species name or cyst morphotype	Molecular clade	Taxonomic order	Sampling site	Depth (m)	Accession number	
						18S	28S
**Cyst 50**	pear-shaped	A	Holacanthida	Iceland Basin	620		JX661004
Ei 59	*Acanthoplegma* sp.	A	Holacanthida	Red Sea	surface	JN811161	JN811246
Pec 14	round	B2	Holacanthida	Mediterranean Sea	surface	JX661014	JX661005
Pec 18	round	B2	Holacanthida	Mediterranean Sea	surface	JX661015	JX661006
Vil 20	round	B2	Holacanthida	Mediterranean Sea	surface	JN811203	JN811306
Vil 39	*Acanthochiasma* sp.	B2	Holacanthida	Mediterranean Sea	surface	JN811206	JN811310
Vil 51	*Acanthochiasma* sp.	B2	Holacanthida	Mediterranean Sea	surface	JN811207	JN811314
Vil 64	*Acanthochiasma* sp.	B2	Holacanthida	Mediterranean Sea	surface	JN811211	JN811318
**Cyst 6**	elongated	B1	Holacanthida	Iceland Basin	160	JX661013	JX660997
**Cyst 43**	elongated	B1	Holacanthida	Iceland Basin	2000	JX661009	JX661003
Ei 68	*Acanthonidium echinoides*	B1	Holacanthida	Red Sea	surface	JN811162	JN811247
Ant 1	*Gigartacon fragilis*	C1	Chauncanthida	Antarctic	surface	KC172858	KC172876
Ant 3	*Gigartacon fragilis*	C1	Chauncanthida	Antarctic	surface	KC172859	KC172877
Ant 8	*Gigartacon fragilis*	C1	Chauncanthida	Antarctic	surface	KC172860	KC172878
Vil 110	*Gigartacon muelleri*	C1	Chauncanthida	Mediterranean Sea	surface	JN811220	JN811328
Pec 12	*Stauracon pallida*	C2	Chauncanthida	Mediterranean Sea	surface	KC172867	KC172886
Pec 13	*Stauracon pallida*	C2	Chauncanthida	Mediterranean Sea	surface	KC172869	KC172887
Oki 33	*Gigartacon fragilis*	C2	Chauncanthida	Pacific Ocean	surface	JN811176	JN811266
Oki 91	*Gigartacon fragilis*	C2	Chauncanthida	Pacific Ocean	surface	JN811199	JN811294
**Cyst 25**	round	C3	Chauncanthida	Iceland Basin	160	JX661007	JX661002
**Cyst 28**	round	C3	Chauncanthida	Iceland Basin	160	JX661008	JX660999
**Cyst 45**	round	C3	Chauncanthida	Iceland Basin	620	JX661010	JX661000
**Cyst 48**	round	C3	Chauncanthida	Iceland Basin	620	JX661011	JX661001
**Adult 20**	unidentified	C3	Chauncanthida	Iceland Basin	160	KC172873	KC172889
Ant 21	*Heteracon* sp.	C3	Chauncanthida	Antarctic	surface	KC172862	KC172880
Ei 71	*Litholophus sp.*	C3	Chauncanthida	Red Sea	surface	JN811163	JN811248
Pec 16	*Heteracon biformis*	C3	Chauncanthida	Mediterranean Sea	surface	KC172870	KC172883
Vil 65	*Heteracon biformis*	C3	Chauncanthida	Mediterranean Sea	surface	JN811212	JN811319
Oki 79	*Litholophus sp.*	C3	Chauncanthida	Pacific Ocean	surface	JN811197	JN811291
Ei 48	*Acanthocyrta haeckeli*	C4	Chauncanthida	Red Sea	surface	JN811157	JN811240
Pec 11	*Heteracon biformis*	C4	Chauncanthida	Mediterranean Sea	surface	KC172866	KC172882
Vil 126	*Heteracon biformis*	C4	Chauncanthida	Mediterranean Sea	surface	JN811223	JN811331
Pec 9	oval	C4	Chauncanthida	Mediterranean Sea	surface	JX661016	JX660996
Vil 131	*Amphiacon denticulatus*	C4	Chauncanthida	Mediterranean Sea	surface	JN811225	JN811333
Ei 47	*Acanthocyrta* sp.	C4	Chauncanthida	Red Sea	surface	JN811156	JN811239
Oki 30	*Gigartacon muelleri*	C4	Chauncanthida	Pacific Ocean	surface	JN811175	JN811265
Oki 23	*Gigartacon muelleri*	C4	Chauncanthida	Pacific Ocean	surface	JN811171	JN811261
Vil 162	oval	C4	Chauncanthida	Mediterranean Sea	surface	JX661017	JX660998
Vil 117	*Gigartacon muelleri*	C4	Chauncanthida	Mediterranean Sea	surface	JN811221	JN811329
Vil 105	*Gigartacon muelleri*	C4	Chauncanthida	Mediterranean Sea	surface	JN811219	JN811327
Vil 52	*Gigartacon muelleri*	C4	Chauncanthida	Mediterranean Sea	surface	JN811208	JN811315
Vil 53	*Gigartacon muelleri*	C4	Chauncanthida	Mediterranean Sea	surface	JN811209	JN811316
Vil 61	*Gigartacon muelleri*	C4	Chauncanthida	Mediterranean Sea	surface	JN811210	JN811317
Pec 5	unidentified	IV	not identified	Mediterranean Sea	surface	KC172865	KC172881
Ros 6	*Trizona brandti*	D	Holacanthida	English Channel	surface	JN811202	JN811298
Oki 47	*Staurolithium* sp.	D	Holacanthida	Pacific Ocean	surface	JN811182	JN811272
Oki 51	*Acanthocolla solidissima*	D	Holacanthida	Pacific Ocean	surface	JN811184	JN811275
Vil 32	*Staurolithium* sp.	D	Holacanthida	Mediterranean Sea	surface	JN811205	JN811308
Oki 28	*Acanthocolla cruciata*	D	Holacanthida	Pacific Ocean	surface	JN811174	JN811264

Oki 77	*Acanthocolla cruciata*	D	Holacanthida	Pacific Ocean	surface	JN811195	JN811289
Oki 4	*Dorataspis loricata*	E	Arthracanthida	Pacific Ocean	surface	JN811166	JN811254
Vil 25	*Coleaspis vaginata*	E	Arthracanthida	Mediterranean Sea	surface	JN811204	JN811307
Oki 74	*Dorataspis loricata*	E	Arthracanthida	Pacific Ocean	surface	JN811194	JN811288
Oki 11	*Lychnaspis giltschi*	E	Arthracanthida	Pacific Ocean	surface	JN811168	JN811257
Oki 49	*Lychnaspis giltschi*	E	Arthracanthida	Pacific Ocean	surface	JN811183	JN811274
Oki 36	*Lychnaspis giltschi*	E	Arthracanthida	Pacific Ocean	surface	JN811177	JN811267
Oki 46	*Lychnaspis giltschi*	E	Arthracanthida	Pacific Ocean	surface	JN811181	JN811271
Oki 45	*Phractopelta sarmentosa*	E	Arthracanthida	Pacific Ocean	surface	JN811180	JN811270
Oki 42	*Phractopelta dorataspis*	E	Arthracanthida	Pacific Ocean	surface	JN811178	JN811268
Oki 73	Larcidium dodecanthum	E	Arthracanthida	Pacific Ocean	surface	JN811193	JN811287
Pec 3	*Larcidium dodecanthum*	E	Arthracanthida	Mediterranean Sea	surface	KC172863	KC172884
Pec 4	*Larcidium dodecanthum*	E	Arthracanthida	Mediterranean Sea	surface	KC172864	KC172885
Vil 161	*Lithoptera* sp.	F	Arthracanthida	Mediterranean Sea	surface	KC172857	KC172875
Ant 17	*Acanthometron* sp.	F	Arthracanthida	Antarctic	surface	KC172861	KC172879
Pec 17	*Acanthostaurus purpurascens*	F	Arthracanthida	Mediterranean Sea	surface	KC172868	KC172888
**Adult 43**	unidentified	F	Arthracanthida	Iceland Basin	620	KC172871	KC172890
Oki 8	*Stauracantha orthostaura*	F	Arthracanthida	Pacific Ocean	surface	JN811167	JN811255
Oki 93	*Stauracantha orthostaura*	F	Arthracanthida	Pacific Ocean	surface	JN811200	JN811295
Oki 43	*Phyllostaurus cuspidatus*	F	Arthracanthida	Pacific Ocean	surface	JN811179	JN811269
Vil 82	*Xiphacantha alata*	F	Arthracanthida	Mediterranean Sea	surface	JN811213	JN811321
Oki 100	*Xiphacantha quadridentata*	F	Arthracanthida	Pacific Ocean	surface	JN811201	JN811297
Ei 51	*Amphistaurus tetrapterus*	F	Arthracanthida	Red Sea	surface	JN811159	JN811242
Ei 53	*Phyllostaurus cuspidatus*	F	Arthracanthida	Red Sea	surface	JN811160	JN811243
Vil 142	*Phyllostaurus cuspidatus*	F	Arthracanthida	Mediterranean Sea	surface	JN811226	JN811334
Vil 85	*Phyllostaurus quadrangulus*	F	Arthracanthida	Mediterranean Sea	surface	JN811214	JN811322
Oki 17	*Xiphacantha quadridentata*	F	Arthracanthida	Pacific Ocean	surface	JN811169	JN811259
Ei 43	*Xiphacantha quadridentata*	F	Arthracanthida	Red Sea	surface	JN811155	JN811238
Vil 95	*Xiphacantha quadridentata*	F	Arthracanthida	Mediterranean Sea	surface	JN811216	JN811324
Ei 24	*Amphilonche elongata*	F	Arthracanthida	Red Sea	surface	JN811151	JN811233
Ei 27	*Amphilonche elongata*	F	Arthracanthida	Red Sea	surface	JN811152	JN811235
Ei 75	*Phyllostaurus siculus*	F	Arthracanthida	Red Sea	surface	JN811164	JN811249
**Adult 52**	unidentified	F	Arthracanthida	Iceland Basin	620	KC172872	KC172891
Oki 21	*Amphibelone heteracanthum*	F	Arthracanthida	Pacific Ocean	surface	JN811170	JN811260
Oki 68	*Amphibelone heteracanthum*	F	Arthracanthida	Pacific Ocean	surface	JN811191	JN811284
Ant 20	Acanthometron sp	F	Arthracanthida	Antarctic	surface	JQ697714	JQ697734
Ant 23	Acanthometron sp	F	Arthracanthida	Antarctic	surface	JQ697715	JQ697735
Ant 24	Acanthometron sp	F	Arthracanthida	Antarctic	surface	JQ697716	JQ697736
Oki24	*Acanthostaurus conacanthus*	F	Arthracanthida	Pacific Ocean	surface	JN811172	JN811262
Oki 65	*Acanthostaurus conacanthus*	F	Arthracanthida	Pacific Ocean	surface	JN811188	JN811281
Ei 49	*Lonchostaurus rombicus*	F	Arthracanthida	Red Sea	surface	JN811158	JN811241
Ei 23	*Lonchostaurus rombicus*	F	Arthracanthida	Red Sea	surface	JN811150	JN811232
N3	Acanthometra fusca	F	Arthracanthida	Mediterranean Sea	surface	KC172856	KC172874
Oki 67	*Acanthometra pellucida*	F	Arthracanthida	Pacific Ocean	surface	JN811190	JN811283
Vil 100	*Acanthostaurus purpurascens*	F	Arthracanthida	Mediterranean Sea	surface	JN811218	JN811326
Vil 127	*Acanthostaurus purpurascens*	F	Arthracanthida	Mediterranean Sea	surface	JN811224	JN811332
Oki 27	*Phyllostaurus siculus*	F	Arthracanthida	Pacific Ocean	surface	JN811173	JN811263
Ant 14	Acanthometron sp	F	Arthracanthida	Antarctic	surface	JQ697713	JQ697733
Ant 10	Acanthometron sp	F	Arthracanthida	Antarctic	surface	JQ697712	JQ697732
Ant 9	Acanthometron sp	F	Arthracanthida	Antarctic	surface	JQ697711	JQ697731
Ant 2	Acanthometron sp	F	Arthracanthida	Antarctic	surface	JQ697708	JQ697729
Ant 7	Acanthometron sp	F	Arthracanthida	Antarctic	surface	JQ697710	JQ697730
