# Supplementary material for: Diversity, Ecology and Biogeochemistry of Cyst-Forming Acantharia (Radiolaria) in the Oceans
Source: PLoS One. 2013 Jan 11;8(1):e53598. doi: 10.1371/journal.pone.0053598 (PMC3543462; doi:10.1371/journal.pone.0053598)
Supplement: Table S2 — Number of V9 tag sequences of the 18S rRNA, assigned to Acantharia and protists, at different stations in big (20–2000 µm) and small (0.8–5 µm) size fraction. (PDF) [file pone.0053598.s004.pdf]

Stations	Size fraction	Surface - Acantharia reads	Surface - Protist reads	Meso - Acantharia reads	Meso - Protist reads
65	Big	4617	1191025	19427	762167
65	Small	21877	1805961		
68	Big	1885	1664986	38428	1062389
68	Small	1672	1333498	19404	1313948
70	Big	884	1709748	35608	1232381
70	Small	14643	1141768	10492	1873138
72	Big	6201	2386096	17488	501735
72	Small	33887	2720703	4426	1327713
76	Big	4112	3917936	81499	220352
76	Small	33998	2296852	9045	1486151
78	Big	11998	4033322	87239	1244972
78	Small	19116	3082893	18105	1335317
85	Big	3885	4053175	1103	1590029
85	Small	1635	3347706	573	1753357
98	Big	9518	2078427	53219	756195
98	Small	3891	1581106	13279	751750
100	Big	5236	3677772	185938	1237608
100	Small	11015	1312311	29343	725876
102	Big	6063	1721153	109426	1027477
102	Small	40533	2450385	21496	792130
	Big	54399	26433640	629375	9635305
Total	Small	182267	21073183	126163	11359380
