# Supplementary material for: Diversity, Ecology and Biogeochemistry of Cyst-Forming Acantharia (Radiolaria) in the Oceans
Source: PLoS One. 2013 Jan 11;8(1):e53598. doi: 10.1371/journal.pone.0053598 (PMC3543462; doi:10.1371/journal.pone.0053598)
Supplement: Table S3 — Comparison of the number of V9 tag sequences assigned to different cysts (>97%) isolated in this study between the photic and the mesopelagic zones. (PDF) [file pone.0053598.s005.pdf]

Depth	Clade	Cyst	65	68	70	72	76	78	85	98	100	102
Photic zone	C4	PEC9	535	2352	134	1335	3146	259	49	213	1101	2241
Mesopelagic	C4	PEC9	1001	587	10	64	47	294	20	81	1597	1374
Photic zone	B1	Cyst 6	35	0	10	10	6	73	172	8	17	52
Mesopelagic	B1	Cyst 6	309	738	7148	122	176	462	163	575	20	290
Photic zone	B2	Vil 20	281	5	6	3440	1322	614	1	17	252	350
Mesopelagic	B2	Vil 20	1203	496	31	68	37	236	7	2804	91	165
